# Practitioner Learning Curve in Fitting Scleral Lenses in Irregular and Regular Corneas Using a Fitting Trial

**DOI:** 10.1155/2019/5737124

**Published:** 2019-01-28

**Authors:** Rute J. Macedo-de-Araújo, Eef van der Worp, José M. González-Méijome

**Affiliations:** ^1^Clinical & Experimental Optometry Research Lab (CEORLab), Center of Physics, University of Minho, Braga, Portugal; ^2^Eye-Contact-Lens Research & Education, Amsterdam, Netherlands

## Abstract

**Purpose:**

To assess the learning curve of a novel practitioner with minor previous experience with scleral lenses (SL) fitting in the initial 156 consecutive fittings in irregular and regular corneas using a fitting trial.

**Methods:**

Prospective dispensing case series involving a total of 85 subjects (156 eyes), 122 eyes with irregular corneas (IC Group) and 34 eyes with regular corneas (RC Group). All lenses were fitted by the same practitioner with minimal previous knowledge and practice on SL fitting. The first 156 consecutive fits were studied to estimate the number of trial lenses required to achieve the optimal fit and the number of reorders required. The results were divided into 8 chronological groups of 20 fittings (eyes) each.

**Results:**

There was a decrease in the number of trial lenses required to achieve the optimal fit from 2.35±0.18 lenses in the first 20 fittings to 1.56±0.13 in the last fittings (p<0.05, Wilcoxon). There were no statistically significant differences between IC and RC groups. Regarding the number of reorders, there was also a decrease from 0.95±0.17 in the first fittings to 0.25±0.11 in the last fittings (p<0.05, Wilcoxon). Thought not statistically significant, there was an increase in the use of toric designs with increasing experience.

**Conclusions:**

Practitioner fitting experience reduced both the number of trial lenses required to achieve the best fit and the number of reorders with time. After the first 60 cases, there was a significant reduction in the trial lenses and reorders necessity.

## 1. Introduction

There is increasing evidence that scleral support rigid gas permeable contact lenses are suitable to compensate a wide range of corneal conditions derived from primary corneal disease and postsurgical complications and even in normal corneas [1–3]. Scleral lenses (SL) have been a matter of research reports in several peer-review journals with an exponential increase in the number of publications over the last years [4]. Although several publications report on long-term outcomes, most recent publications focus on short-term studies with the purpose to evaluate specific features of lens fitting, regarding settling time, [5–8] postlens tear film characteristics [9–11], or the ocular surface response [11–13]

The recent rebirth of SL fitting has been accompanied by a more predictable fitting process, but there is still a significant degree of uncertainty due to the few options of devices to objectively measure anatomical features of the ocular surface beyond the corneal area. Optical coherence tomography (OCT) and scleral topographers are some options that could have an important role during the fitting process; however, they are still not widely used in clinical practice all over the world [4]. Fitting recommendations given by several manufacturers used to consider only the clinical features and the degree of severity of the corneal condition to decide the starting point for fitting. Few studies, however, mention the success rate of the fitting process expressed as the number of lenses needed to accomplish a satisfactory fitting [14]. Understanding this learning curve is relevant for manufacturers and clinicians as this will directly impact the number of lenses required to accomplish a successful fitting. The starting hypothesis for this work is that the number of lenses required to obtain an optimal fit reduces significantly after the initial fitting procedures.

The primary goal of the present study was to analyze the number of trial lenses and reorders required to obtain a satisfactory fitting and to evaluate the learning process from the clinician perspective by evaluating the changes in fitting over the time of enrollment. A secondary goal was to evaluate the differences in the fitting complexity between irregular and normal corneas.

## 2. Methods

### 2.1. Study Design and Subjects

This was a prospective dispensing, case series involving patients with primary corneal ectasia, penetrating keratoplasty, postsurgical ectasia, and regular corneas with high refractive errors between December 2015 and March 2017. The study was conducted at the Clinical and Experimental Optometry Research Lab (CEORLab), at University of Minho (Braga, Portugal). A total of 95 subjects were primarily recruited to participate in a study involving scleral supported contact lens fitting. Lenses were manufactured by Procornea (Eerbeek, Netherlands). Other relevant technical details of the contact lenses are presented in Table 1. Two trial sets were available, one with 16.4 mm (10 trial lenses) and other with 20.0 mm (9 trial lenses) diameter each with different parameters. All the subjects included were new SL wearers or previous SL wearers that were switched to a different lens design.

The subjects were divided into two major groups. One group (IC Group) comprised corneas with primary or secondary ectasias, postpenetrating keratoplasty, and other corneal irregularities due to refractive surgery or others. The second group comprised subjects with regular and healthy corneas (RC Group) that have failed or rejected other forms of vision correction with contact lenses, whether because of comfort or lens stabilization on eye (vision). Only subjects with moderate-to-high refractive errors (myopia > 6.00 D, astigmatism > 2.00 D, and/or hyperopia > 4.00 D) that failed other forms of vision correction were included in RC Group. Subjects with previous ocular surgery were excluded. Subjects of each group were further divided into subgroups for some analysis: Prim.IC included subjects from IC Group with primary ectasia or other conditions not induced by corneal surgeries and Sec.IC included those subjects from IC Group with secondary irregularities due to previous surgeries (corneal irregularities due to refractive surgery, penetrating keratoplasty, intracorneal ring segments implantation, and corneal cross-linking). Subjects from RC Group were separated according to their astigmatism into LA.RC (low astigmatism <2.00 D) and HA.RC (high astigmatism >2.00 D). To be included in the present study patients must have been dispensed with SL and have at least 1 follow-up visit completed (85 subjects).

### 2.2. Measurements

Three repeated measures of corneal topography were done with Medmont E300 (Precision, Vancouver) in each eye in order to assess the severity of each case. Data from simulated keratometry (SimK), which measures the paracentral zone (usually 3 mm) of the anterior surface of the cornea and corneal asphericity (Q) of the flat and steep corneal meridians, were analyzed for each group. High and low contrast visual acuities (HCVA and LCVA, respectively) with ETDRS LogMAR scale charts were measured with habitual correction (HC) and best spectacle correction (BSC). Later, both HCVA and LCVA were also evaluated with SL.

### 2.3. Fitting Procedure Evaluation (Trials and Reorders)

All lenses were fitted by the same practitioner (R.M-A) who was a licensed optometrist with a Master Degree in advanced optometry but without previous clinical experience of scleral lens fitting. Prior to fitting the lenses, she received training on the fitting procedure. Following the recommendations of the declaration of Helsinki, all subjects received information from the study before they accept to participate and signed a consent form. The protocol of the study has been reviewed and approved by the Ethics Subcommittee for Life and Health Sciences of University of Minho.

All the subjects enrolled in this study had to attend several appointments during the follow-up: Baseline, lens dispensing visit, and follow-up visits: 1-, 3-, 6-, and 12-month visits. In this report, only subjects that were dispensed and have at least one follow-up visit were included. At the first appointment (Baseline) SL fitting was done. Fittings were performed using diagnostic fitting sets from Procornea. Subjects that were CL wearers previous to the trial visit were advised not to wear their habitual lenses 3 days before the Baseline appointment. The initial trial lens was determined following manufacturers' guidelines, considering clinical features and the degree of severity of the corneal condition. Practitioner did not use any objective measurement that could aid in the selection of the first trial lens. All lenses were fitted empirically, based on trial and error process, with diagnostic lens sets. The best trial lens should align evenly on the scleral and vault the entire corneal surface and limbus, with a cornea-lens separation of about 300 *μ*m after insertion. Both scleral alignment and cornea-lens separation were subjectively evaluated with slit lamp. If the on-eye fitting of the first trial lens was not satisfactory (i.e., inadequate alignment on the sclera or inadequate cornea-lens separation), a second trial lens was inserted. The process was repeated in both eyes until the practitioner found the best trial lens for each eye. After this, the final fitting assessment was done after a settling time of at least 90 minutes of lens wear [6, 7, 15]. Central and peripheral (limbal) clearance and scleral alignment of trial SL and spherocylinder overrefraction were assessed to order the final lens. The optimal final SL should align evenly on sclera and vault the entire corneal and limbal area with an ideal cornea-lens separation of 100 to 200 *μ*m after settling. The number of trial lenses needed to obtain a satisfactory SL fitting was recorded for each eye.

When the ordered SLs arrived, subjects went to the lens dispensing visit (LDV), where the on-eye fittings were evaluated after lens insertion and after at least 90 minutes of lens wear. If the fitting was not satisfactory, another lens with different parameters was ordered (and was considered a reorder). The number of reorders at LDV (when needed) was recorded for each eye. Then, subjects were also evaluated at several follow-up appointments at 1, 3, and 6 months of lens wear (after LDV); reorders were also recorded at these visits. It was considered a “reorder” whenever it was necessary to order a new lens with different parameters for the same eye. Erroneous shipments and other factors not directly linked to practitioner fitting process were excluded from this analysis.

The number of trial lenses required to prescribe and order the lenses and the number of lenses reordered to the manufacturer at LDV and through the follow-up period were counted and grouped in 8 chronological groups in 20 fittings (eyes), without accounting for the group of the subject (IC or RC Group). Analysis involving the division into the different groups and subgroups was performed without chronological sequence.

Statistical analysis was conducted using SPSS version 24.0 (IBM Co, IL) to compare the number of trial lenses and reorders required between groups and subgroups. Normality of data distribution was analyzed with Shapiro Wilk test in different groups and subgroups. Pairwise comparison between groups or subgroups was done using an independent sample T-Test for normally distributed data and Wilcoxon signed ranks test for nonnormally distributed data. Multiple comparisons to evaluate the effect of time on number of trials and reorders or subject handling and wearing experience were evaluated with ANOVA test for normally distributed data and Kruskal-Wallis test for nonnormally distributed data. The level of statistical significance was set at p<0.05.

## 3. Results

A total of 85 subjects (43 females and 42 males) with a mean age of 34.51±10.41 years were included in this report. Of them, 14 wore lenses in one eye and 71 wore lenses in both eyes representing a total of 156 eyes dispensed with SLs. Since not all fittings were bilateral, there were 5 cases in which both eyes of the same subject fell in different groups, which contributed to increase the chance of final homogenization between all groups. The fittings were divided into 8 groups of 20 fittings, in the chronological order of each fitting, in order to analyze the learning process. The sample was also analyzed separately according to the ocular condition that required the SL fitting in IC Group (irregular corneas, n=122 eyes) and RC Group (high refractive error, n=34 eyes).

Table 2 shows the demographic data of the subjects enrolled in the present report including keratometric data, spherical equivalent refraction, and best corrected visual acuity with habitual correction (HC), best spectacle correction (BSC), and SL. Results are presented separately for irregular (IC Group) and regular corneas (RC Group).

Regarding the results of VA, in IC Group there were statistically significant differences between both HCVA and LCVA with SL when compared to HC (improvement of more than 2 lines, p<0.001). In RC Group those differences were also statistically significant, although clinically insignificant (differences of 2.5 letters, p<0.05). Although HCVA with HC was significantly different between groups, there were no differences between them in HCVA measured with SL, meaning that we can achieve an identical HCVA in healthy and irregular corneas with these kinds of devices. However, there was a statistically significant difference of 1 line of letters in LCVA with SL between the same groups, which reflects that the optical quality in low contrast is significantly worse in subjects with irregular corneas.

### 3.1. Fitting Trials and Reorders

**Figure 1:**
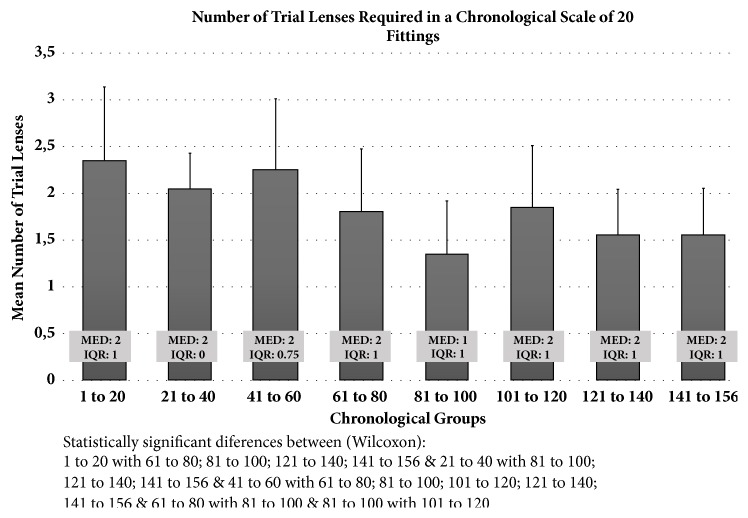
Number of trial lenses required to achieve the best fit. Data is presented in a chronological scale of 20 fittings. Bars represent the mean number of trial lenses and respective standard deviation. Boxes show the median (MED) and interquartile range (IQR) for each chronological group.

Figure 1 presents the number of lenses required during the fitting trial in a chronological scale of 20 fittings. According to the chronological order of the fittings we observed a tendency to decrease the number of lenses required to achieve the optimal fitting to be dispensed, a decrease from a mean of 2.35±0.79 in the first 20 chronological fittings to a mean of 1.56±0.50 in the last 16 fittings (p<0.05, Wilcoxon). The number of trial lenses required began to be statistically significantly lower than the first 20 fittings after fittings 61 to 80 (p<0.05, Wilcoxon).

The mean number of lenses trialed to arrive to the final dispensing SL in the trial visit was 1.85±0.71 lenses, being 1.84±0.69 for IC Group (range between 1 and 4 trial lenses) and 1.88±0.77 for RC Group (range between 1 and 4 trial lenses). When both groups were compared, there were no statistically significant differences between them regarding the number of trial lenses needed to achieve the best fit (p=0.970, Mann–Whitney U test). By further dividing the sample into subgroups, more lenses, on average, were required for Sec.IC (irregular corneas submitted to surgeries, 1.98±0.72 lenses) than for Prim.IC (1.78±0.67 lenses), but without statistically significant differences between them (p=0.149, Mann–Whitney U test), and more trial lenses for HA.RC (with astigmatism >2.00 D, 1.96±0.82 lenses) than for LA.RC (1.63±0.52 lenses), also without statistically significant differences (p=0.413, Mann–Whitney U test).

**Figure 2:**
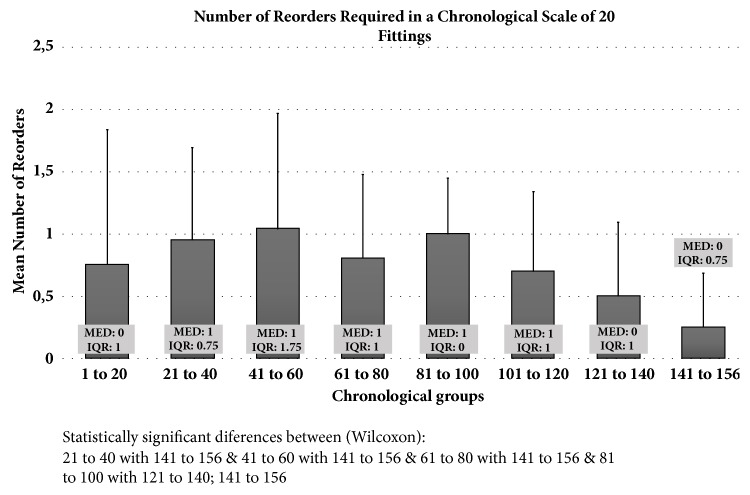
Number of reorders required after the first lens dispensed. Data is presented in a chronological scale of 20 fittings. Bars represent the mean number of trial lenses and respective standard deviation. Boxes show the median (MED) and interquartile range (IQR) for each chronological group.

Figure 2 presents the number of reorders required in a chronological scale of 20 fittings. According to the chronological order of the fittings there was a decrease in the reorders required (a reduction of an average of 0.95±0.74 at fittings 21 to 40 to 0.25±0.43 in fittings 141 to 156 (p<0.05, Wilcoxon), meaning a reduction of an average of almost 1 reorder per subject to 1 reorder per 4 subjects on the last fittings. The number of lens reorders began to decrease after fitting number 60 (p<0.05, Wilcoxon).

The average number of reorders needed was 0.76±0.77 lenses, being 0.73±0.76 for the IC Group (range between 0 and 4 trial lenses) and 0.88±0.81 for the RC Group (range between 0 and 3 trial lenses), without statistically significant differences between them (p=0.303, Mann–Whitney U test). By further dividing the sample into subgroups, the Sec.IC required statistically more reorders to achieve the best fit (0.98±0.92) when compared to Prim.IC (0.60±0.63) (p<0.05, Mann–Whitney U test). But when comparing the mean number of reorders between LA.RC and HA.RC (1.00±0.76 and 0.85±0.83, respectively), there were no statistically significant differences (p=0.537, Mann–Whitney U test). However, 73.3% of the reorders performed on RC Group were done on HA.RC subgroup, with also as higher number of fittings (Table 3). Table 3 shows the number of lenses ordered to the manufacturer to accomplish a satisfactory fitting. Results are presented separately for each group and each subgroup according to the nature of the irregular astigmatism (primary ectasia or surgically induced in IC Group) and regular astigmatism (low or moderate-to-high astigmatism (≥ 2.00 DC) in RC Group). The visit when the reorders were needed is also shown.

Most of the reorders performed were due to inadequate sagittal height (more than 30% in both groups), poor vision (23.6%, IC Group), and a combination between poor vision and inadequate fit (33.3%, RC Group). An important issue is that about 10% of the subjects of each group required a reorder because of lens discomfort, although the fitting seemed satisfactory. Most of the changes were done in the landing zone of the lens, namely, refitted with toric designs, which resulted in improved comfort. Another important factor is the number of lenses that broke (5 in IC Group and 1 in RC Group); 4 of them broke during mechanical handling disinfection (rubbing the lenses), 1 lens fell on the floor during application, and 1 lens suffered an* in situ* breakage after being hit by a high speed projectile, but without compromise to the corneal surface [16]. More than 70% of the reorders needed in both groups were made in the lens dispensing visit; however, there were also 3 subjects that required a reorder after 3 months of lens wear: 2 because of lenses that broke and 1 because of continuous discomfort.

### 3.2. Back and Front Toric Designs

**Figure 3:**
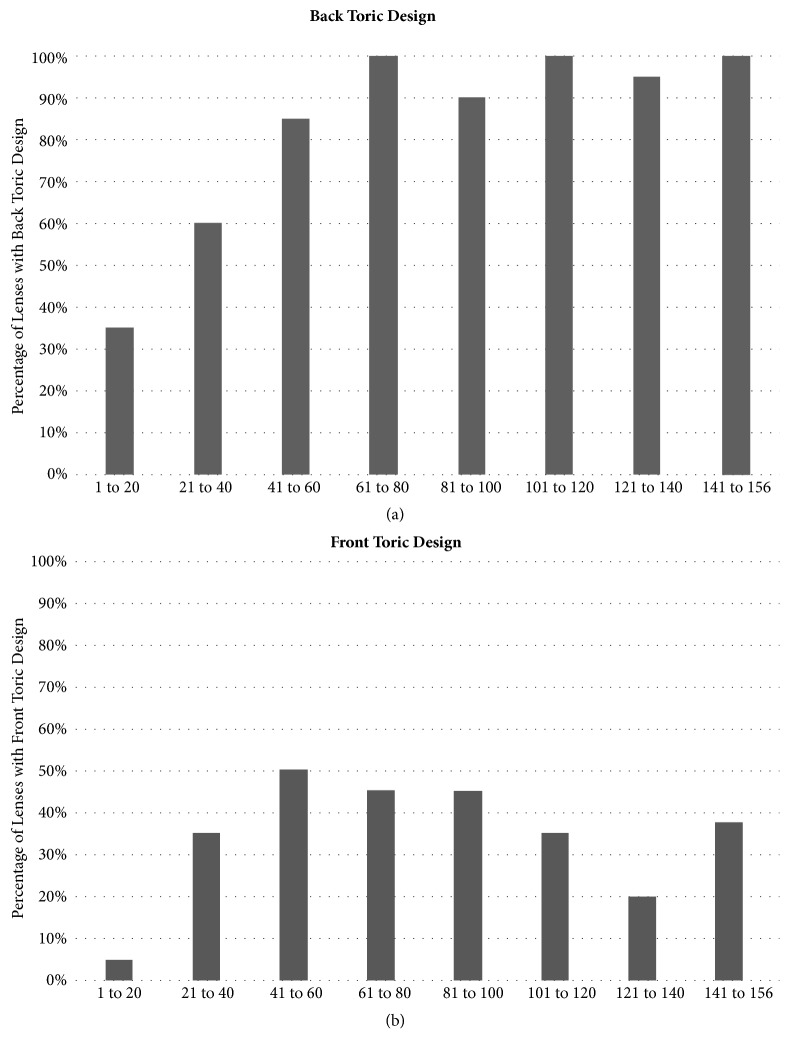
Percentage of lenses with back toric lens design (a) and front toric lens design (b) required. Data is presented in a chronological scale of 20 fittings.

Figure 3(a) shows the percentage of SL with landing zone toric designs required in a chronological scale of 20 fittings. According to the chronological order of fittings, an increase in the number of landing zone toric lens designs required was observed. The number of landing zone toric designs duplicated between the first 20 fittings (35%) and fittings numbers 41 to 60 (97%). In IC Group, 85% of the total lenses fitted were toric and 74% of RC Group were toric.  Figure 3(b) shows the percentage of SL with front toric designs required in a chronological scale of 20 fittings. From the 156 total number of fittings, 53% required front toric lens designs. The value of astigmatism that was required ranged from -0.50 D to -2.00 D (mean of -0.90±0.28 D).

## 4. Discussion

Several studies have already proven the visual efficacy of SL for different eye conditions, from normal/regular shaped corneas to the more challenging corneal irregularities [1, 3, 4, 17]. Although many experts state that there is a steep learning curve in fitting these devices, there are no publications regarding the complexity and the learning curve in fitting SL for a beginner practitioner. This is the first study that confirms that SLs can be successfully fitted by practitioners with minor previous training using fitting trials method. However, there is a training period after which there is a reduction in both trial lenses needed and reorders performed to achieve the best SL that subjects can comfortably wear successfully.

The recently published results of SCOPE online survey on demographic and prescribing patterns of SL fitters [18] revealed that the number of practitioners fitting SL has increased during the past decade. From the total 989 respondents, 19% reported to have fitted at least 5 patients with SL. From the practitioners that completed the entire survey (n=678), 65% reported that they have fitted 50 or fewer patients during their career and 21% reported that they had fitted only 10 or fewer patients. A limited number of experienced fitters were identified (13%), who reported to have fitted more than 200 patients. Despite the valuable information drawn by this study about the number of SL fitters and their demographics and academic background and how many patients are actually wearing SL, there are no results of how many lenses have those two distinct groups of practitioners required to achieve the best fit for each patient.

In the present study, we identified how many trial lenses were required to achieve the optimal lens to be dispensed and how many reorders were required after the first dispensed lenses, and how this learning curve evolves over time in a novel practitioner without previous experience in SL fitting. Figure 1 shows a decrease in the number of trial lenses required to achieve the optimal fit to be dispensed. An average of 2.35±0.79 lenses per eye were necessary in the first fittings, which reduced to 1.50 trial lenses or less after the first 61 to 80 fittings (eyes) were accomplished. Although the time spent in each trial visit was not recorded in this study, this reduction of 1 trial lens per eye could have a significant positive effect in the chair time required. In this study, the practitioner and the devices used in each trial visit were the same, so this improvement of 1 lens per eye reveals an improvement in practitioner's clinical judgment with time. The findings shown in this study might be affected by more challenging or easier to fit cases that might appear at any time during the chronological course of this study. However, the relatively large sample recruited and the uniformity in inclusion and exclusion criteria should minimize this factor and contribute to a uniform distribution of cases with different degrees of difficulty in reaching the final fitting. A preliminary analysis comparing refraction, topography, and quality of vision parameters between the 8 chronological groups was conducted. Despite statistically significant differences between the 8 groups in some topography and quality of vision parameters, there was not a pattern that suggests a chronological change in easiness or difficulty between groups. In fact, these parameters are not necessary related to the difficulty of fitting. Regarding proportion of patients without or with previous surgery or different grades of ectasia severity or no ectasia, they were evenly distributed between the 8 chronological groups.

Although there are no studies in peer-review literature reporting the potential improvement of practitioner skills over time in fitting SL (learning curve), there are few studies reporting the mean number of trial lenses or lenses ordered per eye to achieve the best fit during the fitting process. Schornack et al. [19] found an average of 1.5 lenses ordered per eye and an average of 2.8 visits to complete the fitting process in a sample of 19 patients with keratoconus (30 eyes), and Gemules [20] reported an average of 1.7 attempts per patient for the 9 patients enrolled in the study. Studies with corneal rigid gas permeable contact lenses (RGP) reported a mean number of 2.3 trial lenses to achieve the best fit (range: 1 to 5 lenses per eye) [21] and other study reported an average of 1.73 lenses [22]. According to our results, a mild-experienced SL fitter would need an average of 1.50 trial lenses per eye, which is lower than the values provided by those studies for RGP corneal lenses.

The differences between IC and RC groups on the mean number of trial lenses required to achieve the best fit (1.84±0.69 and 1.88±0.77, respectively) were not statistically significant. By further dividing our results in subgroups, postsurgical corneas (Sec.IC) required more trial lenses than those with primary ectasia. Corneas with high astigmatism (HA.RC) also required more trial lenses to achieve the best fit. Although there were no statistically significant differences between them, this means that irregular corneas submitted to surgeries or corneas with high astigmatism could be more challenging to fit in some cases. By personal experience of the practitioner, those corneas that underwent specific surgeries (like penetrating keratoplasty and intracorneal ring implantation) or those with high astigmatic corneas (namely, with limbus-to-limbus high toricity) are often more challenging to fit. Possible explanations to this include the more asymmetric corneal surface in the postsurgical corneas and the more asymmetric scleral shape associated with the highly toric corneas (in the RC Group). Although there is lack of consensus in this regard, some clinical observations revealed that, when the corneal astigmatism is higher and congenital in nature, the sclera could also have the same magnitude and orientation of toricity [23, 24]. Also, Marcus Ritzmann et al. [25] did not find a strong association between the orientation and magnitude of corneal astigmatism and scleral toricity in normal corneas, except for some eyes. The authors also concluded that higher corneal astigmatism (>2.00D) could be associated with scleral toricity. Other studies found that the scleral topography of irregular and regular shaped corneas have differences, which could have a direct impact on SL fitting, namely when choosing the best landing zone geometry for the different eyes [26–28].

Regarding the reorders needed during the fitting process, we found a 40% optimal fit rate with the first lens ordered. To our knowledge, this has never been established for SL in the peer-reviewed literature. A work presented at GSLS 2018, which analyzed the first 150 fits in a normal clinical practice, reported that 27.9% of the subjects completed the fit with no changes to the initial lens order [14]. In corneal RGPs there are also significantly different reported rates: Romero-Jimenez et al. reported an optimal fit rate of 77% [21] and Betts et al. [29] reported 33% using the same lenses; discrepancies between studies were justified by differences in the methodology. In our sample, 48% of the total sample required 1 lens exchange, 9% required 2 lens exchanges, and 4% required 3 or 4 lens reorders. On average, the mean number of lens ordered per eye was 1.76±0.77, which is similar to the reported values of a recent work by Adeline Bauer (1.70 lenses per eye). [14]

Although it seemed to have an increase in the number of reorders in the first fittings, we rapidly see a tendency to decrease (Figure 2). That early increase in the number of reorders was attributed to the augmented complexity of the cases after fitting number 20. After these initial 20 fittings, the experience of the practitioner shows a higher rate of back surface toricity prescription, which could require some additional reorders in the beginning as the practitioner gets familiar with the clinical impact of different changes in fitting parameters.

The differences between both groups on the mean number or reorders (0.73±0. 76 and 0.88±0.81, respectively) were small and with no statistically significant difference. When further dividing into subgroups, and similar to what we concluded about trial lenses, Sec.IC subgroup needed more reorders than Prim.IC (p<0.05), which corroborates our thoughts about the complexity of fitting those corneas that underwent some surgeries. Controversial to the findings on the mean number of trial lenses required in each subgroups of RC Group, no statistically significant differences were found (mean of 1.00±0.76 on LA.RC and 0.85±0.83 in HA.RC). In fact, there is a large difference in the number of subjects of each subgroup (8 in LA.RC and 26 in HA.RC), but we can also see that 73.4% of the total number of reorder were from the subgroup of corneas with high astigmatism. As said before, the clinical feeling of the practitioner was that high astigmatic corneas were more complex to fit. In 3 fittings of HA.RC Group it was required to order a different trial lens because none of the lenses from the trial set fitted correctly the scleral shape (landing zone) because of high scleral toricity.

Regarding the prescribing pattern of more specific designs, 83% of the total fits have toric landing zone designs (85% in IC Group and 74% in RC Group). This is in accordance with Gregory DeNayer et al. [30] findings, that 94.3% of the 140 eyes analyzed with a scleral topographer showed nonspherical-like scleral shapes, meaning that the vast majority of the eyes analyzed could benefit from nonspherical landing zone geometries to perfectly align with the scleral shape. There was also an increase in using central and landing zone toric lens designs with increasing experience: 35% of the first 20 fittings had landing zone toric design, which increased to 97% in the last fittings. Once again, the authors recognize that these results should be analyzed with caution. Indeed, eyes requiring SL with toric landing zones or with internal astigmatism requiring central toricity could present at any time during the clinical trials, so it is difficult to address that this could be only related to a change in the practitioner skills.

There are some factors that could be seen as limitations of the study. First, only 1 practitioner/fitter was evaluated to assess the learning curve: other practitioners could learn faster or slower and this will have a direct impact on the study findings. Second, the results of this study are limited to the fitting of SLs using trial sets with the same characteristics of the ones used in this study. Current fitting approaches by most practitioners use a similar procedure what allows to apply current results to most fitting protocols. However, other designs and manufacturers might not replicate exactly the present results and they need to be independently assessed. Also, technologies such as OCT and scleral topographers are increasingly being used during SL fittings, which could aid during the fitting process and consequently decrease the number of trial lens and reorders. Also, techniques derived from corneal topography, like the ones described in another study by the same authors [24], might also aid during the fitting process, but they need to be prospectively assessed. In addition, other approaches could be used to assess the cornea-lens separation (central corneal clearance, CCC), such as the use of optic biometers or using an image processing software (like ImageJ) to measure CCC more objectively than with slit lamp alone [31]. Altogether, these, could have a direct impact on the number of trial lenses and reorders to the manufacturer.

The authors' decision to use both eyes of each subject (when applicable) was because 78% of the total sample were irregular corneas and it is well established that the majority of these conditions are asymmetric in nature. These asymmetries will influence the lens fitting, namely, the lens sagittal height for each eye. In addition, SLs land on conjunctiva, so the anatomy of the eye beyond the corneal borders has an important role in the fitting process. Despite some degree of correlation in refractive error or corneal power between both eyes (which are not necessary related to the difficulty of SL fitting process), there were poor correlations considering the geometry of the lens landing zone in the two groups (r=0.364 IC Group and r=0.333 RC Group, Spearman). Considering the clinical experience of the authors that, despite similarities that might be present between both eyes of the same subject, the level of complexity of the fitting process is not so straightforward; specific adjustments are often required. Further limitations include the asymmetric number of patients/eyes in the different subgroups. However, altogether, the present study presents one of the largest case series recently published.

In summary, we have observed that contemporary scleral supported rigid gas permeable lenses can be fitted in most cases of moderate-to-severe ocular corneal defects and regular shaped corneas by practitioners with minimal previous training. After the first fittings, a novel practitioner would be able to significantly reduce the number of trial lenses and reorders to the manufacturer.

## Figures and Tables

**Table 1:** Characteristics of the mini- and full-scleral lenses trial sets used in the present study.

Parameter	Mini-scleral lens	Full-scleral lens
Material	Boston XO (hexafocon A)	Boston Equalens II (oprifocon A)

Dk (ISO/Fatt)	100	85

Central Thickness (-3.00 D)	0.25 mm	0.45 mm

Diameter	From 15.20 to 18.00 mm in 0.40 mm steps	From 18.00 to 24.50 mm in 0.50 mm steps

Back Optic Radius	8.20 mm (from 7.00 to 9.40 mm in 0.20 steps)	From 7.20 mm to 9.80 mm in 0.10 mm steps

Power	Sphere +20.00 D to -25.00 D in steps of 0.25 D; Front cyl -0.50D to -3.00D in steps of 0.25D; Axis 0 to 180 degrees in steps of 1 degree	Sphere +30.00 D to -30.00 D in steps of 0.25 D; Front cyl -0.50D to -3.00D in steps of 0.25D; Axis 0 to 180 degrees in steps of 1 degree

Refractive Index	1.415	1.423

Hardness	81/112 (Shore/ Rockwell)	114 (Rockwell)

Density	1.27	1.24

Contact Angle (deg.)	49	30

Sagittal height	From 0.25 to 6.75 in 0.25 steps	From 2.47 to 5.07 in 0.10 steps

Peri-Factor / Sclera Opening	From -8 to +8 in steps of 1	From 11.50 to 17.25 in 0.25 steps

Toricity (difference in peri-factor)	From 1 to 6 in steps of 1	From 1 to 4 in steps of 1

**Table 2:** Demographic data of the patients analyzed in each clinical subgroup included in the present report.

		Total	IC Group	RC Group	p
No. Subjects		85	67 (79%)	18 (21%)	-

No. Eyes Fitted		156	122 (78%)	34 (22%)	-

Gender		43 female (51%) 42 male (49%)	31 female (46%) 36 male (54%)	12 female (67%) 6 male (33%)	-

Subgroup (No. Fittings)			Prim.IC: 80 (66%) Sec.IC: 42 (34%)	LA.RC: 8 (24%) HA.RC: 26 (76%)	-

Age (years)		34.51±10.41 (range: 16 to 65)	35.54± 10.45 (range: 16 to 65)	30.67±9.91 (range: 18 to 35)	0.080+

SimK Flat (D)		43.93±5.51	44.20±6.19 [range: 17.03 to 62.92]	42.99±1.62 [range: 39.45 to 46.01]	**<0.001** **∗**

SimK Steep (D)		47.29±6.04	47.78±6.74 [range: 18.83 to 65.38]	45.58±1.71 [range: 43.20 to 45.58]	**<0.001** *∗*

Q Flat		-0.65±0.53	-0.71±0.58 [range: -2.89 to +0.84]	-0.43±0.19 [range: -0.86 to -0.11]	**<0.05**+

Q Steep		-0.17±0.69	-0.26±0.71 [range: -1.59 to +2.24]	0.14±0.51 [range: -0.32 to 1.64]	**<0.05** *∗*

HC (No. Eyes)	Glasses	73	45	28	-
	Soft CL	19	13	6	-
	RGP	20	20	0	-
	Hybrid	13	13	0	-
	SL	11	11	0	-
	N/P	20	20	0	-

HCVA w/ HC		+0.30±0.30	+0.34±0.31 [range: -0.18 to +1.40]	+0.16±0.21 [range: -0.10 to +0.60]	**<0.001** *∗*

LCVA w/ HC		+0.54±0.32	+0.62±0.33 [range:+ 0.10 to +1.80]	+0.31±0.18 [range: +0.08 to +0.9]	**<0.001** *∗*

BSC	M (D)	-3.64±3.63	-3.24±3.23 [range: -15.00 to +3.00]	-4.94±4.57 [range: -13.13 to +1.88]	0.078*∗*
	J0 (D)	0.23±1.02	-0.04±0.92 [range: -1.38 to +3.29]	1.09±0.89 [range: -0.44 to +3.20]	**<0.001** *∗*
	J45 (D)	0.20±1.13	0.23±1.26 [range: -3.20 to +3.50]	0.12±0.61 [range: -1.10 to 2.09]	0.820*∗*

HCVA w/ BSC (LogMAR scale)		+0.26±0.27	+0.31±0.28 [range: -0.10 to +1.00]	+0.11±0.17 [range: -0.10 to +0.60]	**<0.001** *∗*

LCVA w/ BSC (LogMAR scale)		+0.51±0.30	+0.58±0.29 [range: +0.10 to +1.80]	+0.29±0.18 [range: +0.08 to +0.90]	**<0.001** *∗*

HCVA w/ SL (LogMAR scale)		+0.07±0.15	+0.08±0.15 [range: -0.18 to +0.62]	+0.06±0.15 [range: -0.20 to +0.48]	0.650+

LCVA w/ SL (LogMAR scale)		+0.32±0.18	+0.34±0.18 [range: +0.02 to +0.94]	+0.24±0.15 [range: +0.04 to +0.60]	**<0.05** *∗*

IC: Irregular Cornea; RC: Regular Cornea; ♀ female; ♂ male; PrimIC: primary ectasia; SecIC: secondary ectasia; LA.RC: Low Astigmatism; HA.RC: High Astigmatism; HC: Habitual Correction; BSC: Best Spectacle Correction; HCVA: High Contrast Visual Acuity; LCVA: Low Contrast Visual Acuity; SL: Scleral Lenses; N/P: No prescription; Q: corneal asphericity; (+) Independent T-test; (*∗*) Mann-Whitney U independent samples.

**Table 3:** Number of lenses reordered in each group (irregular and regular cornea) and subgroup (surgical/nonsurgical and low and high astigmatism).

		TOTAL fittings (n=156)	IC Group			RC Group		
			**TOTAL (n=122)**	Prim.IC (n=80)	Sec.IC (n=42)	**TOTAL** **(n=34)**	LA.RC ≤ 2.00 D (n=8)	HÁ.RC ≥ 2.00 D (n=26)
Cause of reorder	**Inadequate Sagittal Height** **∗**	**36**	**27**	12	15	**9**	3	6
		**(30.3**%**)**	**(30.3**%**)**	(13.5%)	(16.9%)	**(30.0**%**)**	(10.0%)	(20.0%)
	**Inadequate Landing Zone**	**7**	**5**	2	3	**2**	0	2
		**(5.9**%**)**	**(5.6**%**)**	(2.2%)	(3.4%)	**(6.7**%**)**	(0%)	(6.7%)
	**Poor Vision**	**23**	**21**	16	5	**2**	0	2
		**(19.3**%**)**	**(23.6**%**)**	(18.0%)	(5.6%)	**(6.7**%**)**	(0%)	(6.7%)
	**Discomfort**	**12**	**9**	5	4	**3**	3	0
		**(10.1**%**)**	**(10.1**%**)**	(5.6%)	(4.5%)	**(10.0**%**)**	(10.0%)	(0%)
	**Poor Vision + Fit** ^Δ^	**21**	**11**	6	5	**10**	2	8
		**(17.6**%**)**	**(12.4**%**)**	(6.7%)	(5.6%)	**(33.3**%**)**	(6.7%)	(26.7%)
	**Fit** ^Δ^	**14**	**11**	4	7	**3**	0	3
		**(11.8**%**)**	**(12.4**%**)**	(4.5%)	(7.9%)	**(10.0**%**)**	(0%)	(10.0%)
	**Lens Broke**	**6**	**5**	3	2	**1**	0	1
		**(5.0**%**)**	**(5.6**%**)**	(3.4%)	(2.2%)	**(3.3**%**)**	(0%)	(3.3%)

Visit of reorder	**Lens Dispensing Visit (V0)**	**85**	**64**	34	30	**21**	5	16
		**(71.4**%**)**	**(71.9**%**)**	(38.2%)	(33.7%)	**(70**%**)**	(16.7%)	(53.3%)
	**1 week visit**	**8**	**6**	3	3	**2**	0	2
		**(6.7**%**)**	**(6.7**%**)**	(3.4%)	(3.4%)	**(6.7**%**)**	(0%)	(6.7%)
	**1 month visit (V1)**	**19**	**12**	6	6	**7**	3	4
		**(16.0**%**)**	**(13.5**%**)**	(6.7%)	(6.7%)	**(23.3**%**)**	(10.0%)	(13.3%)
	**3 month visit (V2)**	**4**	**4**	3	1	**0**	0	0
		**(3.4**%**)**	**(4.5**%**)**	(3.4%)	(1.1%)	**(0**%**)**	(0%)	(0%)
	**>3 month visit**	**3**	**3**	2	1	**0**	0	0
		**(2.5**%**)**	**(3.4**%**)**	(2.2%)	(1.1%)	**(0**%**)**	(0%)	(0%)

	**Total number of reorders**	**119**	**89**	48	41	**30**	8	22
		**(100**%**)**	**(100**%**)**	(53.9%)	(46.1%)	**(100**%**)**	(26.7%)	(73.3%)

n is the number of fittings; *∗*both increased and decreased sagittal height; ^Δ^Poor Fit = both sagittal height and landing zone parameters are inadequate.

## Data Availability

The data used to support the findings of this study are available from the corresponding author upon request.
